# Antibody profiling identifies novel antigenic targets in spinal cord injury patients

**DOI:** 10.1186/s12974-016-0713-5

**Published:** 2016-09-13

**Authors:** Ilse Palmers, Elke Ydens, Eric Put, Bart Depreitere, Helma Bongers-Janssen, Peter Pickkers, Sven Hendrix, Veerle Somers

**Affiliations:** 1 Biomedical Research Institute and transnationale Universiteit Limburg, School of Life Sciences, Hasselt University, Martelarenlaan 42, Hasselt, Belgium; 2 Department Neurosurgery, Jessa Hospital, Stadsomvaart 11, Hasselt, Belgium; 3 Division of Experimental Neurosurgery and Neuroanatomy, Katholieke Universiteit Leuven and University Hospitals Leuven, Herestraat 49, Leuven, Belgium; 4 Adelante Rehabilitation, Zandbergsweg 111, Hoensbroek, The Netherlands; 5 Department of Intensive Care Medicine, Radboud University, Nijmegen Medical Centre, Geert Grooteplein Zuid 10, Nijmegen, The Netherlands

**Keywords:** Antigenic targets, Antibody repertoire, Serological antigen selection, cDNA phage display library, Spinal cord injury

## Abstract

**Background:**

Recent evidence implicates antibody responses as pivotal damaging factors in spinal cord injury (SCI)-induced neuroinflammation. To date, only a limited number of the antibody targets have been uncovered, and the discovery of novel targets with pathologic and clinical relevance still represents a major challenge.

**Methods:**

In this study, we, therefore, applied an unbiased, innovative and powerful strategy, called serological antigen selection (SAS), to fully identify the complex information present within the antibody repertoire of SCI patients.

**Results:**

We constructed a high-quality cDNA phage display library derived from human spinal cord tissue to screen for antibody reactivity in pooled plasma samples from traumatic SCI patients (*n* = 10, identification cohort). By performing SAS, we identified a panel of 19 antigenic targets to which the individual samples of the plasma pool showed antibody reactivity. Sequence analysis to identify the selected antigenic targets uncovered 5 known proteins, to which antibody reactivity has not been associated with SCI before, as well as linear peptides. Immunoreactivity against 9 of the 19 novel identified targets was validated in 41 additional SCI patients and an equal number of age- and gender-matched healthy subjects. Overall, we found elevated antibody levels to at least 1 of the 9 targets in 51 % of our total SCI patient cohort (*n* = 51) with a specificity of 73 %. By combining 6 of these 9 targets into a panel, an overall reactivity of approximately half of the SCI patients could be maintained while increasing the specificity to 82 %.

**Conclusions:**

In conclusion, our innovative high-throughput approach resulted in the identification of previously unexplored antigenic targets with elevated immunoreactivity in more than 50 % of the SCI patients. Characterization of the validated antibody responses and their targets will not only provide new insight into the underlying disease processes of SCI pathology but also significantly contribute to uncovering potential antibody biomarkers for SCI patients.

## Background

Neuroinflammation is a key process in spinal cord injury (SCI) pathophysiology that can persist for months or even years after primary trauma. Its strong dual character in SCI underscores the need for an in-depth understanding of the complex neuroinflammatory processes [1–3]. While the responses mediated by innate immune cells and T cells have been extensively studied, the exact contribution of B cells and the humoral response that is triggered after SCI remains elusive. In experimental SCI, B cells accumulated in the injured spinal cord, formed follicle-like structures and contributed to aggravated tissue damage [4]. B cell-knockout mice displayed a highly improved neurological recovery and a markedly less pronounced neuropathology after SCI compared to wild-type (WT) mice [5]. SCI-induced B cell activation culminated in the production of pathologic and central nervous system (CNS)-reactive antibodies which were released in the blood stream and migrated to the lesion [4, 5]. Passive transfer of serum antibodies from SCI mice into naïve/uninjured mice showed that these SCI-induced antibodies participated in neuroinflammatory responses and exerted a degenerative effect in the spinal cord by causing cell death and sustained neurological dysfunction [4, 5]. A proteomics study on spinal cord tissue in mice indicated that more than 50 different proteins were targeted by antibodies after SCI; however, up to now, only glutamate receptor 2/3 and nuclear antigens have been described as antibody targets in SCI models [4, 6].

In human SCI pathology, increased antibody responses to a number of CNS proteins (glial fibrillary acidic protein (GFAP), myelin basic protein (MBP)), glycoproteins (myelin-associated glycoprotein (MAG)), glycolipids (GM_1_ gangliosides) and nuclear antigens have been detected. The antibody titers to several of those myelin components remained elevated until years after the initial trauma [7–13]. Interestingly, the titers of these antibodies seemed to correlate with clinical parameters (e.g. SCI complications) [7, 8, 10]. Identification of the full spectrum of antigenic targets of the SCI-induced antibody responses is indispensable to develop passive and active antibody-based therapeutics or diagnostic and prognostic agents.

To date, only antibody responses to target antigens described in other CNS disorders have been investigated in SCI pathology. This hypothesis-driven approach yielded only limited success in identifying clinically relevant antibody responses. The heterogeneous nature of the SCI population further underscores the importance of an innovative and unbiased approach, to fully explore the complex information present in the antibody repertoire of SCI patients. Serological antigen selection (SAS) is a powerful high-throughput method based on complementary DNA (cDNA) phage display and subsequent selection on patient antibodies which enables the identification of a broad profile of antigenic targets [14–16]. By using a cDNA phage display library derived from the target tissue, a disease-relevant system that is fully representative for the heterogeneity present within the target tissue is created. This approach allows the identification of not only known proteins but also unknown or uncharacterized proteins. SAS has been successfully applied in our lab to identify an elaborate panel of novel relevant antibody responses for various diseases, such as multiple sclerosis, clinically isolated syndrome and rheumatoid arthritis [17–19]. We have demonstrated not only the pathologic in vivo relevance of these antibody responses but also their diagnostic and prognostic potential [20–22].

In the present study, we constructed a high-quality cDNA expression library from human spinal cord tissues which allowed rapid isolation of novel antigenic targets and identified generic antibody responses by using plasma samples from SCI patients. Subsequently, detailed serological characterization of these antibody responses was assessed in SCI patients and healthy controls.

## Methods

### Patient samples

Peripheral blood was collected from traumatic and pathologic SCI patients in Jessa Hospital (Hasselt, Belgium), University Hospitals Leuven (Leuven, Belgium), Adelante Rehabilitation Centre (Hoensbroek, The Netherlands), Hospital East-Limburg (Genk, Belgium), Antwerp University Hospital (Edegem, Belgium), Radboud University Medical Centre (Nijmegen, The Netherlands) and Academic Hospital Maastricht and General Hospital Turnhout (Turnhout, Belgium). Samples of traumatic SCI patients were taken at hospitalization (T0) or 3 weeks after injury (T1). Samples of pathologic SCI patients (e.g. stenosis, spinal disc herniation, compression caused by bleeding) were collected preoperatively (T0) and 3 weeks after surgery (T1). Patients with pre-existing autoimmune disorders were excluded from the study. Written informed consent was acquired from all participants after approval by the Medical ethics committee Hospital East Limburg (B371201317091), Adelante (54-14/CK/JM) and Academic Hospital Maastricht (METC13-4-079). Blood samples were centrifuged for 10 min at 400*g*. Plasma was collected and centrifuged for 10 min at 1500*g*. After processing, plasma samples were aliquoted and stored at −80 °C. Samples were processed and stored in collaboration with the University Biobank Limburg (UBiLim) and Biobank University Hospitals Leuven. A total of 51 SCI patients and 49 age- and gender-matched healthy controls were involved in the study. Plasma samples from healthy controls were collected via UBiLim.

### Construction of a hSC cDNA phage display library

Commercially obtained poly A+ RNA (size range of 0.2–10 kb, Clontech, Saint-Germain-en-Laye, France) from spinal cord tissue of 18 Caucasians (ages 25–63 years) was converted to double-stranded cDNA with EcoRI and XhoI adapters by using the Superscript Choice System for cDNA synthesis kit (Life Technologies, Gent, Belgium) as described previously [23]. Purified cDNA inserts were directionally ligated into our pVI phage display vectors, pSPVIA, pSPVIB and pSPVIC, each representing 1 of 3 different reading frames [16]. Ligation mixtures were used to transform *Escherichia coli* (*E.coli*) TG1 cells (Lucigen, Middleton, USA) by electroporation to obtain human spinal cord (hSC)-pSPVIA, hSC-pSPVIB and hSC-pSPVIC libraries.

### Serological antigen selection procedure

The serological antigen selection (SAS) procedure was performed as described previously [16, 19]. In brief, an immunotube (Nunc, Roskilde, Denmark) was coated overnight at 4 °C with 10 μg/ml rabbit anti-human immunoglobulin G (IgG, Dako, Glostrup, Denmark) in coating buffer (0.1 M sodium hydrogen carbonate, pH 9.6). After washing with 0.1 % (*v*/*v*) phosphate-buffered saline-Tween20 (PBS-T, 50 mM Tris, 150 mM sodium chloride, pH 7.5) and PBS, the immunotube was blocked with 2 % (*w*/*v*) skimmed milk powder in PBS (MPBS) for 2 h at room temperature (RT). Plasma samples of 10 traumatic SCI patients were pooled and pre-adsorbed against *E. coli* and phage components, as described previously [16]. For the first selection round, equal numbers of phage particles from each hSC cDNA phage display library (hSC-pSPVI-A, hSC-pSPVI-B and hSC-pSPVI-C) were pre-incubated with the pre-adsorbed SCI plasma pool in 2 % MPBS for 1.5 h at RT on a rotating platform [24]. After washing the immunotube, the pre-incubated phage-plasma mix was transferred to the coated tube on a rotating platform, followed by standing conditions at RT. Non-bound phage were removed by extensive washing of the immunotube with 0.1 % PBS-T and PBS. Bound phage were eluted by adding 100 mM triethylamide (Sigma-Aldrich, Bornem, Belgium) to the immunotube for 10 min on a rotating platform and neutralized with 1 M Tris-HCl (pH 7.4). The output of each selection round was amplified by infection of *E. coli* TG1 bacteria and plated on ×2 YT agar plates containing ampicillin and glucose (16 g/l bacto-tryptone, 10 g/l yeast extract, 5 g/l NaCl, 15 g/l bacto-agar, ampicillin at 100 μg/ml, and glucose at 2 %). Five consecutive selection rounds were performed. To identify enriched cDNA clones, individual colonies were selected and insert cDNA fragments were amplified with vector primers binding adjacent to the cDNA insert followed by restriction enzyme digestion (BstNI (Roche Diagnostics, Vilvoorde, Belgium) and NspI (NEB, Leiden, The Netherlands)). Enriched cDNA products representing identical cDNA clones were selected and identified by sequencing of the corresponding cDNA phage insert. Amino acid sequences of identified clones were compared to public protein databases of the National Center for Biotechnology Information (NCBI) with BLAST analysis.

### Phage ELISA

Antibody reactivity levels of individual plasma samples against selected phage clones were measured by phage enzyme linked immunosorbent assay (ELISA). Ninety-six-well flat-bottom plates (Greiner Bio-One, Wemmel, Belgium) were coated overnight at 4 °C with 5 μg/ml anti-M13 antibody (GE Health care, Diegem, Belgium) in coating buffer. Plates were washed twice with PBS and blocked with 5 % MPBS for 2 h at 37 °C, while shaking. After washing three times with 0.1 % PBS-T and once with PBS, polyethylene glycol-purified phage displaying the candidate antigen (7 × 10^11^ colony-forming units/ml) or empty phage (negative control) were added and incubated for 1 h at 37 °C under static conditions followed by 30 min at RT, while shaking. Plates were washed, and plasma samples (1/100 in 5 % MPBS) were incubated for 1 h at 37 °C under static conditions followed by 30 min at RT, while shaking. Washing steps were repeated, and horseradish peroxidase human IgG-Fc fragment cross-adsorbed antibody (1/50,000, Bethyl laboratories, Montgomery, USA) was added for 1 h shaking at RT. 3,3′,5,5′-Tetramethyl-benzidine dihydrochloride (TMB, Thermo Scientific, Erembodegem, Belgium) solution was added after washing the plates, and the reactions were incubated in the dark for 11 min. The colour reaction was stopped by adding 1.8 N H_2_SO_4_. Optical density (OD) signals were measured at 450 nm in a Tecan plate reader (Tecan, Männedorf, Switzerland). Samples were considered positive when the general reactivity (OD (specific phage)/OD (empty phage)) was higher than 1.5 and the OD-value of a specific signal was above 0.1. Samples were tested in duplicate in a single ELISA experiment, and experiments were performed independently for at least two times. Polyreactive samples (reactive to the empty phage) were excluded from the analysis.

### Statistical analysis

Statistical analysis was performed using GraphPad Prism 6 XML. Fisher’s exact test was applied for the analysis of associations between the presence of antibody reactivity directed to particular antigenic targets or panels of targets and SCI patients. A *p* value of <0.05 was considered statistically significant.

## Results

### Antibody profiling of SCI plasma samples using SAS

A high-quality hSC cDNA phage display library was generated from human spinal cord tissue of 18 Caucasians. Human spinal cord cDNA fragments were cloned into the pVI phage display vectors in three reading frames, resulting in a total library size of 2.44 × 10^6^ independent clones. The spinal cord cDNA inserts ranged from 350 to 2000 base pairs. Details of the performed quality controls are described in [23]. Altogether, these results showed that the hSC cDNA phage display library had a high quality and diversity. Subsequently, this hSC cDNA phage display library was screened for antibody reactivity using a plasma pool consisting of 10 randomly selected traumatic SCI patients (identification cohort; mean age 48 years (range from 27 to 85 years); Table 1). In the identification cohort, both samples collected at hospitalization (i.e. within 48 h after traumatic injury; T0) and 3 weeks after injury (T1) were included. The majority of the patients were men (9/10) and had a cervical level injury (6/9). Both injury severity and type (compression, contusion, fracture or laceration) were highly diverse within the pool. By using a heterogeneous plasma pool, the general patient population was represented and the detection of patient-specific immunoreactivity was limited.

**Table 1:** Characteristics of traumatic SCI patients used for the SAS procedure (identification cohort)

Patient	Time of sampling (T)^a^	Age (years)	Gender^b^	Location of injury^c^	Type of tSCI^d^
SCI.1	T0	27	M	T5	Laceration
SCI.2	T0	29	M	T6-10	Fracture
SCI.3	T0	37	M	C4-6	Contusion
SCI.4	T0	43	M	C4-5	Contusion
SCI.5	T0	54	M	C6	Fracture
SCI.6	T0	67	M	NA^e^	Compression
SCI.7	T1	33	M	C6-T2	Contusion
SCI.8	T1	29	M	C4	Contusion
SCI.9	T1	73	F	C1	Compression
SCI.10	T1	85	M	C6	Contusion

^a^ *T0* at hospitalization, *T1* 3 weeks after injury

^b^ *F* female, *M* male

^c^ *C* cervical, *T* thoracic

^d^ *tSCI* traumatic spinal cord injury

^e^Not available

To analyse the overall antibody reactivity profile in SCI patients, SAS was used. Five consecutive selection rounds were performed. After characterization of the selection output, a total of 38 enriched phage clones were selected which represent putative antigenic targets present after SCI.

### Antibody reactivity is increased in plasma of traumatic SCI patients

**Fig. 1:**
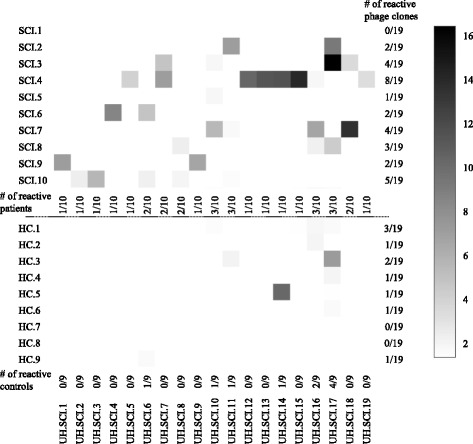
Increased antibody reactivity in plasma of traumatic SCI patients. Antibody reactivity toward the identified targets is increased in the individual traumatic SCI samples used in the SAS procedure (*n* = 10) compared to healthy controls (*n* = 9). Samples were considered positive when the general reactivity (OD (specific phage)/OD (empty phage)) was higher than 1.5 and the specific signal was above 0.1. Antibody levels in positive samples are shown in a *grey scale*. Low antibody levels are indicated in *light grey* (ratio reactivity (OD (specific phage)/OD (empty phage)) is 2); high antibody levels are indicated in *dark grey* (ratio reactivity (OD (specific phage)/OD (empty phage)) ranged from 12 to 16). Samples were analysed in duplicate in a single ELISA experiment, and experiments were performed independently for at least 2 times

To confirm that the enriched phage clones were selected based on specific interactions with SCI patient antibodies and to characterize the reactivity toward the identified clones, a pilot screen was performed on the individual plasma samples from the 10 traumatic SCI patients used in the SAS procedure (identification cohort) and an equal number of age- and gender-matched healthy controls. As shown in Fig. 1, 19 phage clones showed antibody reactivity in 9 of the 10 traumatic SCI plasma samples, while no or low reactivity was detected in healthy controls, which demonstrates that SCI-related antibodies were identified (Fig. 1). The remaining phage clones displayed no reactivity in traumatic SCI patients or reactivity in the control samples. SCI-reactive antigenic targets were annotated an UH.SCI.number (Hasselt University, SCI, clone number). Based on the level and abundance of the antibody responses, our results show that traumatic SCI patients have an increased reactivity toward the selected antigenic targets compared to the healthy control group.

### Identity of antigens targeted by SCI-related antibodies

To gain insight into the antibody responses that are present after SCI, the targets of the selected antibody responses were identified using sequencing and the amino acid sequences of the 19 antigenic targets were compared to public protein databases using BLAST analysis of NCBI (Table 2). Five of the selected antigenic targets (UH.SCI.2, UH.SCI.5, UH.SCI.6, UH.SCI.7 and UH.SCI.19) encoded parts of known proteins: 26S proteasome non-ATPase regulatory subunit 4 (PSMD4), glyceraldehyde-3-phosphate dehydrogenase (GAPDH), myeloma-overexpressed gene 2 (MYEOV2), protein S100-B (S100B) and adipocyte enhancer-binding protein 1 (AEBP1). The remaining antigenic targets resulted from the expression of novel cDNA sequences, out of frame expression of known cDNAs or normally untranslated messenger RNA (mRNA) regions (e.g. 3′UTR regions). These expressed peptides likely form epitopes which structurally mimic in vivo antigens (mimotopes) and showed partial homology to proteins which might be implicated in brain disorders (e.g. ras/Rap GTPase-activating protein (SYNGAP1), to different known proteins (e.g. zinc finger protein 148 (ZN148) and receptor tyrosine-protein phosphatase eta (PTPRJ)) and to proteins with unknown function (e.g. transmembrane protein 236 (TMEM236)) [25]. Upon close examination of the different targets, it becomes clear that the identified antigenic targets are highly diverse with regard to their size (4 to 120 amino acids), sequence composition and amino acid characteristics (hydrophobic, polar or charged). This is also illustrated by the reactivity profile detected in an individual traumatic SCI patient (SCI.4), who shows specific and strong reactivity toward several targets (UH.SCI.5, UH.SCI.7, UH.SCI.12-15 and UH.SCI.19) which are diverse in sequence and structure characteristics. Altogether, our results demonstrate that antibody responses to a broad range of antigenic targets are present in SCI patients.

**Table 2:** Target identity of the SCI-related antibody responses

Clone	Translated cDNA product	Size (amino acids)	Homology on protein level
Coding			
UH.SCI.2	TEEDDYDVMQDPEFLQSVLENLPGVDPNNEAIRNAMGSLASQATKDGKKDKKEEDKK*	57	(56/56) 26S proteasome non-ATPase regulatory subunit 4 (PSMD4)
UH.SCI.5	HRVVDLMAHMASKE*	14	(13/13) glyceraldehyde-3-phosphate dehydrogenase (GAPDH)
UH.SCI.6	TFPEGAGPYVDLDEAGGSTGLLMDLAANEKAVHADFFNDFEDLFDDDDIQ*	50	(49/50) myeloma-overexpressed gene 2 protein (MYEOV2)
UH.SCI.7	HEFFEHE*	7	(7/7) protein S100-B (S100B)
UH.SCI.19	PVETYTVNFGDF*	12	(11/11) adipocyte enhancer-binding protein 1 (AEBP1)
Non-coding			
UH.SCI.1	IKTVTSQ*	7	(6/7) transmembrane protein 236 (TMEM236)
UH.SCI.3	TNNFITSNKN*	10	(6/6) calcium-activated chloride channel regulator 2 (CLCA2)
UH.SCI.4	PLKDIIDNI*	9	(6/6) Di-*N*-acetylchitobiase precursor (CTBS) (6/8) poly(A)-specific ribonuclease PARN (PARN) (6/6) TBC1 domain family member 2B (TBC1D2B)
UH.SCI.8	NSSKTYVGTSDKVQTPSRDLGCPLGSHCSLSLLT*	34	No significant similarity found
UH.SCI.9	NSELLSNKSALHKFIKYAFWI*	21	(12/19) E3 ubiquitin-protein ligase HACE1 (HACE1)
UH.SCI.10	PFFTVPIPRPGA*	12	(8/8) F-box only protein 42 (FBXO42)
UH.SCI.11	EFFDNSRKVDD*	11	(6/8) Ras/Rap GTPase-activating protein SynGAP (SYNGAP1)
UH.SCI.12	NSKHSLKS*	8	(6/7) zinc finger protein 148 (ZNF148)
UH.SCI.13	EDKT*	4	(4/4) Tax1-binding protein 3 (TAX1BP3)
UH.SCI.14	TQEGAGSERGVITTF*	15	(10/14) receptor-type tyrosine-protein phosphatase eta (PTPRJ)
UH.SCI.15	TEGKEQERSDKDE*	13	(7/9) Espin (ESPN)
UH.SCI.16	QGEDKISVY*	9	(6/6) Midasin (MDN1)
UH.SCI.17	NSLHSLLGQKNND*	13	(10/13) Zinc finger protein 28 (ZNF28)
UH.SCI.18	QRLFRQSSSQELLGCGSKTLMGGEGWLLEEGRSQTGQTVLLTPSPAQRALPLWPLLQTPALTHTPLGLHSFALKDLPKSPFPCLASPLRRERYLQNWVGGMSMNCPSIWDMLHQSERENK	120	No significant similarity found

*stop codon

### Validation of the identified antibody responses in SCI patients

Next, we addressed whether antibody reactivity toward the 19 novel antigenic targets was also present in a larger confirmatory cohort of SCI patients. Therefore, we screened 41 additional SCI patients from which samples collected within the same time range as the identification cohort samples were available. This allowed us to validate the identified antibody responses in a similar phase after injury. After large-scale screening of the SCI patient samples and age- and gender-matched healthy controls, immunoreactivity against 9 of the 19 novel identified targets was validated. For 19/41 (46 %) additional SCI patients, antibody reactivity was detected toward at least 1 of the 9 targets. The remaining targets were excluded because patient reactivity could not be confirmed in our validation cohort or high reactivity was detected in control samples.

Our total SCI cohort (identification and validation cohort) consisted of 51 SCI patients (82 % males, mean age 57 ± 16 years) and contained both traumatic and pathologic SCI patients. Furthermore, 49 age- and gender-matched controls (65 % males, mean age 55 ± 17 years) were included in the screening (Table 3). Of the total SCI cohort, 26/51 (51 %) patients showed antibody reactivity to at least 1 of the 9 validated targets (Table 4). The frequency of antibody reactivity against the individual targets within the SCI patients ranged from 4 to 20 %. The highest reactivity and SCI specificity was demonstrated using a panel of 6 targets. Immunoreactivity toward this SCI patient-associated antigen panel could be detected in approximately half of the SCI patients (47 %) with an associated specificity of 82 % (*p* = 0.00145, Table 4). For 1 of the individual antigenic targets, UH.SCI.9, a significant association was found between the presence of antibody reactivity and SCI patients (*p* = 0.0134; overall reactivity 12 %; specificity 100 %, Table 4). Interestingly, while only traumatic SCI patients were included in the selection of SCI-related antibody responses, also 46 % of the pathologic SCI patients showed increased immunoreactivity toward this panel of 6 novel identified targets (Table 4).

**Table 3:** Characteristics of the study populations

Cohort	Time of sampling (*T*)^a^	Mean age (in years)	Gender (F/M)^b^	AIS^c^	Location of injury^d^	tSCI/pSCI ratio^e^
Patients *n* = 51	T0–T1	57	9/42	A-E	C-S	25/26
Healthy controls *n* = 49	–	55	17/32	–	–	–

^a^ *T0* at hospitalization, *T1* 3 weeks after injury or surgery

^b^ *F* female, *M* male

^c^American Spinal Injury Association Impairment Scale

^d^ *C* cervical, *S* sacral

^e^ *tSCI* traumatic spinal cord injury, *pSCI* pathologic spinal cord injury

**Table 4:** Antibody reactivity in the SCI cohort and age- and gender-matched healthy controls

Clone	Identification cohort (*n* = 10)	Validation cohort (*n* = 41)	Total cohort (*n* = 51)			HC^c^ (*n* = 49)	Fisher’s exact test (*p* value)^d^
			tSCI^a^ (*n* = 25)	pSCI^b^ (*n* = 26)	Total (*n* = 51)		
UH.SCI.1	1/10 (10 %)	3/41 (7 %)	2/25 (8 %)	2/26 (8 %)	4/51 (8 %)	2/49 (4 %)	ns
**UH.SCI.2**	1/10 (10 %)	4/41 (10 %)	2/25 (8 %)	3/26 (12 %)	5/51 (10 %)	1/49 (2 %)	ns
**UH.SCI.7**	2/10 (20 %)	2/41 (5 %)	2/25 (8 %)	2/26 (8 %)	4/51 (8 %)	2/49 (4 %)	ns
UH.SCI.8	2/10 (20 %)	1/41 (2 %)	2/25 (8 %)	1/26 (4 %)	3/51 (6 %)	2/49 (4 %)	ns
**UH.SCI.9**	1/10 (10 %)	5/41 (12 %)	3/25 (12 %)	3/26 (12 %)	6/51 (12 %)	0/49 (0 %)	0.0134
**UH.SCI.11**	3/10 (30 %)	7/41 (17 %)	5/25 (20 %)	5/26 (19 %)	10/51 (20 %)	5/49 (10 %)	ns
UH.SCI.12	1/10 (10 %)	1/41 (2 %)	1/25 (4 %)	1/26 (4 %)	2/51 (4 %)	0/49 (0 %)	ns
**UH.SCI.13**	1/10 (10 %)	1/41 (2 %)	1/25 (4 %)	1/26 (4 %)	2/51 (4 %)	1/49 (2 %)	ns
**UH.SCI.15**	1/10 (10 %)	4/41 (10 %)	3/25 (12 %)	2/26 (8 %)	5/51 (10 %)	1/49 (2 %)	ns
Total cohort	7/10 (70 %)	19/41 (46 %)	13/25 (52 %)	13/26 (50 %)	26/51 (51 %)	13/49 (27 %)	0.0073
Panel of 6 targets (bold)	6/10 (60 %)	18/41 (44 %)	12/25 (48 %)	12/26 (46 %)	24/51 (47 %)	9/49 (18 %)	0.00145

^a^ *tSCI* traumatic spinal cord injury

^b^ *pSCI* pathologic spinal cord injury

^c^ *HC* healthy control

^d^ *ns* not significant

**Fig. 2:**
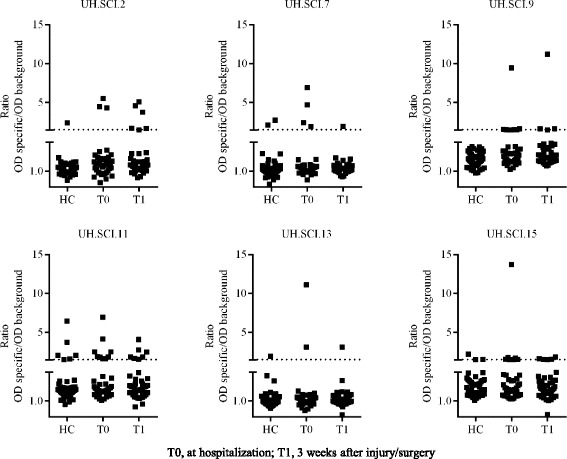
Antibody reactivity in SCI samples collected at hospitalization and 3 weeks after injury or surgery. Immunoreactivity toward the panel of 6 novel identified antigenic targets is found in samples collected at hospitalization (collected within 48 h and maximum 4 days after injury, T0) and 3 weeks after injury or surgery (T1). Antibody reactivity is illustrated as the ratio (OD (specific phage)/OD (empty phage)). Samples were considered positive when the general reactivity (OD (specific phage)/OD (empty phage)) was higher than 1.5 and the specific signal was above 0.1. Samples were analysed in duplicate in a single ELISA experiment, and experiments were performed independently for at least 2 times

Whether or not the presence of these identified antibody responses after SCI was determined by the time point of sampling, we compared the antibody reactivity in patient samples collected at hospitalization (T0) and at 3 weeks after injury (traumatic SCI) or surgery (pathologic SCI) (T1) (Fig. 2). Within the SCI cohort, both traumatic and pathologic SCI patients showed antibody reactivity at hospitalization and 3 weeks after injury or surgery. Notably, most antibody-positive patients exhibit reactivity at both time points of sampling. Altogether, immunoreactivity toward 9 novel identified antigenic targets was validated in both traumatic and pathologic SCI patients, which highlights the relevance of these novel identified antibody responses and their targets in inflammatory mechanisms present after damage to the spinal cord.

## Discussion

In the present study, the antibody signature of SCI patients was explored by using a powerful and unbiased high-throughput strategy, called SAS. By pooling plasma of 10 patients with highly diverse lesion characteristics (location, type and severity of the lesion), we analysed the general antibody reactivity profile present in the heterogeneous SCI patient population. Our innovative and unbiased approach resulted in the identification of 19 distinct antigenic targets with increased reactivity in SCI patients compared to healthy controls. We confirmed reactivity toward 9 of the 19 targets in an additional cohort of 41 SCI patients, including both traumatic and pathologic SCI patients, and an equal number of age- and gender-matched healthy controls. Antibody reactivity against these 9 novel targets was found in 51 % of the total SCI patient cohort. By combining 6 of these 9 targets into a panel, an overall reactivity of approximately 50 % could be maintained while increasing the specificity to 82 %.

We here report immunoreactivity toward various novel antigenic targets in SCI patients, identified via a powerful unbiased approach. A proteomics study using spinal cord tissue already indicated that over 50 distinct antigenic targets are present after SCI in mice [4, 6]. By using a tissue-specific cDNA library containing cDNA sequences encoding proteins with a nervous system-specific expression pattern or proteins involved in pathologic processes triggered after SCI, we created a relevant strategy to identify novel antibody targets in SCI [23]. Our results indicate that this highly diverse antibody profile is also present in SCI patients. Furthermore, we are the first to thoroughly address the identity of the various antigenic targets after SCI. Previously, hypothesis-driven studies only demonstrated elevated antibody responses toward MBP, MAG, GFAP, GM1 ganglioside and galactosylceramidase in sera of SCI patients, and we now extend the antibody profile with responses directed against S100B, GAPDH, PSMD4, AEBP1 and MYEOV2 [7–13]. The first novel identified target, S100B, is a dimeric calcium-binding protein localized predominantly in astroglial and Schwann cells, and increased levels of this protein have been found in CSF and serum of SCI patients [26–29]. While antibody responses directed against S100B were shown in CNS pathologies such as traumatic brain injury (TBI), they have not been reported in SCI patients before [30, 31]. S100B protein was detected in a very early stage after SCI with peak levels detectable already 6 h after injury [32, 33]. Interestingly, in this study, antibody responses toward UH.SCI.7 (S100B) were already present in samples taken within 48 h after injury. Target UH.SCI.5 partly encodes GAPDH which is a well-studied multidimensional protein involved in the homeostatic regulation and is often referred to as a “housekeeping” protein [34, 35]. In stroke models, nuclear GAPDH cascades are triggered leading to posttranscriptional modifications and allowing GAPDH to play a crucial role in brain damage [35, 36]. Because of the multifunctional involvement of GAPDH in the brain, it is not surprising that GAPDH might play a role in SCI pathology [35, 36]. PSMD4 is a non-ATPase subunit of the proteasomal 19S regulator which plays a key role in the recognition and processing of ubiquitylated proteins for proteolysis, and AEBP1 may have a proinflammatory function and is involved in apoptosis and cell survival [37, 38]. The function of MYEOV2, on the other hand, is still unknown. Furthermore, 14 peptide sequences were identified which resulted from the expression of novel cDNA sequences, out of frame expression of known cDNAs or untranslated mRNA regions (e.g. 3′UTR regions) and probably comprise mimotopes. Overall, we identified antibody responses against a broad panel of targets which further underlines the strength of using an unbiased approach such as SAS.

During immune maturation, B cells are not exposed to the variety of unique CNS antigens expressed on neurons, oligodendrocytes, microglia and astrocytes causing a lack of tolerance against these CNS-specific proteins. Although evidence suggests that the blood-spinal cord barrier (BSCB) is more permeable than the blood-brain barrier (BBB), under normal conditions, an intact blood-CNS barrier protects neurons and glial cells from antibodies that cross-react with neurological tissue [39]. However, when the blood-CNS barrier is damaged, these circulating antibodies can infiltrate the CNS with the potential destruction of the neurologic tissue. A recent study showed that the lack of S100B compromises the BBB and allows access of CNS-reactive antibodies to the brain which generates pathological changes [40]. Upon SCI, the BSCB permeability is compromised as long as 56 days [41]. Antibodies against S100B might however prolong BSCB permeability resulting in a continued neuroinflammatory response. Yet, even after reconstitution of BSCB integrity, processes such as adsorptive endocytosis, active transport across the blood-CNS barrier and local antibody production can result in antibody accumulation in the injured spinal cord [4].

The antibody responses toward the novel identified targets were detected both in samples collected at hospitalization and 3 weeks after injury or surgery. The mechanisms and different phases of antibody production after SCI have been investigated in SCI mouse models [4, 5]. It was suggested that after SCI and damage of the blood spinal cord barrier, CNS antigens are released into the bloodstream and drain into peripheral lymphoid tissues where they encounter the cells of the adaptive immune system. Newly formed IgG antibodies are typically formed 3 weeks after the insult [42]. The formation of new antibodies in SCI is supported by Ankeny et al. who showed that serum IgM levels are increased in SCI mice during the first 2 weeks after injury, while serum IgG_2a_ levels are delayed until week 2 and remained elevated up to 42 days post injury [4]. Besides newly formed immune responses, resting or memory B cells can become primed or re-activated by cognate antigens or polyclonal stimuli which has been suggested as a mechanism for antibody responses to CNS proteins in SCI pathology. Our results imply that both newly formed antibody responses and molecular mimicry mechanisms might be relevant in human SCI pathology.

Interestingly, while only traumatic SCI patients were used in the identification of the novel antibody responses, increased reactivity toward the identified antigenic targets was also evident in nearly half of the pathologic SCI patients. While traumatic SCI is an acute event, pathologic SCI is often a more chronic process. Since antibodies are stable, become amplified in the immune response and have a long half-life, their presence over time in pathology is not unexpected and highlights the relevance of these antibody responses in common inflammatory processes that are triggered after injury to the spinal cord.

Besides the potential relevance of the identified targets and their corresponding antibody responses in SCI pathophysiology, the discovery of these novel SCI-related antibody responses is also highly relevant from a clinical viewpoint. We here report individual antigenic targets with an overall reactivity varying from 4 to 20 %, and with high specificity ranging from 90 to 100 %, which are comparable to the previously reported antibody reactivity toward GM1 gangliosides, GFAP, MAG and MBP [7–9, 11, 12]. One of our novel identified antigenic targets (UH.SCI.9) was significantly associated with SCI patients. By combining 6 antigenic targets in a SCI patient-associated panel, we found relevant immunoreactivity in 47 % of the SCI patients with a 82 % specificity. Multiplexing of inflammatory biomarkers in SCI pathology has been shown to be a valuable strategy to significantly improve the sensitivity rate while maintaining a high specificity [28]. Previous studies showed that enhanced antibody levels correlated with complications in SCI patients [7, 8, 10]. So far, association of the novel antibodies with clinical parameters could not be determined as our SCI patient population is heterogeneous and a limited sample size was screened. Gender-related influences on the outcome after SCI have been reported with a better recovery in females. The improved recovery has, at least partly, been attributed to the regulation of the SCI-induced neuroinflammatory response by sex hormones [43, 44]. In our cohort, analyzing the validated antibody responses based on gender did not show any sex-related differences in antibody reactivity against the novel identified targets. Still, as antibodies have been suggested to represent better biomarkers than their antigen counterparts, and we find increased antibody responses already very early after the injury, the investigation of their potential as a prognostic biomarker for SCI patients is highly desirable [45–47].

## Conclusions

In summary, the antibody reactivity profile after SCI in humans was explored by using an unbiased and powerful high-throughput technology based on phage display, which resulted in the identification of 9 novel antigenic targets with elevated immunoreactivity in SCI patients compared to healthy controls. By combining 6 of these 9 antigenic targets into a panel, an overall reactivity of nearly 50 % could be detected with a specificity of 82 %. Further characterization of both the SCI-related antibody responses and the corresponding targets will provide more insight into the role of the humoral immune component within SCI-induced neuroinflammation and their clinical relevance.

## Abbreviations

AEBP1: Adipocyte enhancer-binding protein 1
BBB: Blood-brain barrier
BSCB: Blood-spinal cord barrier
CNS: Central nervous system
*E.coli*: *Escherichia coli*
ELISA: Enzyme linked immunosorbent assay
GAPDH: Glyceraldehyde-3-phosphate dehydrogenase
GFAP: Glial fibrillary acidic protein
h: Hour
hSC: Human spinal cord
MAG: Myelin-associated glycoprotein
MBP: Myelin basic protein
Min: Minutes
MPBS: Skimmed milk powder in PBS
MYEOV2: Myeloma-overexpressed gene 2
NCBI: National Center for Biotechnology Information
OD: Optical density
PBS-T: Phosphate-buffered saline-Tween20
PSMD4: 26S proteasome non-ATPase regulatory subunit 4
PTPRJ: Receptor tyrosine-protein phosphatase eta
RT: Room temperature
S100B: Protein S100-B
SAS: Serological antigen selection
SCI: Spinal cord injury
SYNGAP1: Ras/Rap GTPase-activating protein
T0: At hospitalization
T1: 3 weeks after injury
TMEM236: Transmembrane protein 236
TMB: 3,3′,5,5′-Tetramethyl-benzidine dihydrochloride
UBiLim: University Biobank Limburg
UH.SCI.number: Hasselt University, SCI, clone number
WT: Wild type
ZN148: Zinc finger protein 148
